# ‘It was hell in the community’: a qualitative study of maternal and child health care during health care worker strikes in Kenya

**DOI:** 10.1186/s12939-021-01549-5

**Published:** 2021-09-23

**Authors:** Michael L. Scanlon, Lauren Y. Maldonado, Justus E. Ikemeri, Anjellah Jumah, Getrude Anusu, Sheilah Chelagat, Joann Chebet Keter, Julia Songok, Laura J. Ruhl, Astrid Christoffersen-Deb

**Affiliations:** 1 Indiana University Center for Global Health, 702 Rotary Circle, Suite RO 101, Indianapolis, IN USA; 2 Academic Model Providing Access to Healthcare (AMPATH), Eldoret, Kenya; 3 Department of Medicine and Pediatrics, Massachusetts General Hospital, Boston, MA USA; 4 Department of Child Health and Paediatrics, College of Health Sciences, School of Medicine, Moi University, Eldoret, Kenya; 5 Department of Medicine, Indiana University School of Medicine, Indianapolis, IN USA; 6 Department of Obstetrics and Gynaecology, University of British Columbia, Vancouver, Canada; 7 Department of Obstetrics and Gynaecology, University of Toronto, Toronto, Canada

**Keywords:** Health care worker strike, Maternal child health, Kenya, Qualitative study

## Abstract

**Background:**

Health care workers in Kenya have launched major strikes in the public health sector in the past decade but the impact of strikes on health systems is under-explored. We conducted a qualitative study to investigate maternal and child health care and services during nationwide strikes by health care workers in 2017 from the perspective of pregnant women, community health volunteers (CHVs), and health facility managers.

**Methods:**

We conducted in-depth interviews and focus group discussions (FGDs) with three populations: women who were pregnant in 2017, CHVs, and health facility managers. Women who were pregnant in 2017 were part of a previous study. All participants were recruited using convenience sampling from a single County in western Kenya. Interviews and FGDs were conducted in English or Kiswahili using semi-structured guides that probed women’s pregnancy experiences and maternal and child health services in 2017. Interviews and FGDs were audio-recorded, translated, and transcribed. Content analysis followed a thematic framework approach using deductive and inductive approaches.

**Results:**

Forty-three women and 22 CHVs participated in 4 FGDs and 3 FGDs, respectively, and 8 health facility managers participated in interviews. CHVs and health facility managers were majority female (80%). Participants reported that strikes by health care workers significantly impacted the availability and quality of maternal and child health services in 2017 and had indirect economic effects due to households paying for services in the private sector. Participants felt it was the poor, particularly poor women, who were most affected since they were more likely to rely on public services, while CHVs highlighted their own poor working conditions in response to strikes by physicians and nurses. Strikes strained relationships and trust between communities and the health system that were identified as essential to maternal and child health care.

**Conclusion:**

We found that the impacts of strikes by health care workers in 2017 extended beyond negative health and economic effects and exacerbated fundamental inequities in the health system. While this study was conducted in one County, our findings suggest several potential avenues for strengthening maternal and child health care in Kenya that were highlighted by nationwide strikes in 2017.

## Background

Maternal and neonatal mortality remains a significant public health issue in Kenya [1]. The country did not achieve targets for reductions in maternal and child mortality by 2015 set by the Millennium Development Goals, and progress has been inequitably distributed [2]. In 2017, the maternal mortality ratio was estimated to be 342 per 100,000 live births [3], and only about 60% of women attended four or more antenatal care (ANC) visits and delivered with a skilled birth attendant according to the most recent Demographic and Health Survey in 2014 [4]. Recent government initiatives have aimed to improve access to and utilization of maternity services [5], including a new scheme launched in 2017 called Linda Mama that covers maternity services, including antenatal care (ANC), delivery, and postnatal care, under the national health insurer, the National Hospital Insurance Fund [6]. These policies have been associated with moderate increases in ANC coverage and delivery with a skilled birth attendant, but significant demand- and supply-side barriers remain, particularly for women who are poorer and reside in more rural communities [7–11].

There have been frequent strikes by health care workers in Kenya in the past decade, which likely have significant but under-investigated short- and long-term impacts on maternal and child health care and services. Public sector health care workers have used strikes to protest a wide range of issues, including low pay, poor working conditions, corruption, and breaking of collective bargaining agreements by the government, among others [12]. Strikes have accompanied significant reforms in Kenya, most notably expanded labor rights for workers (including health care workers) to form unions and devolution of health services from the national government to 47 newly created County governments under the new 2010 Constitution [13]. The most protracted strikes occurred in 2017 when public sector physicians launched a 100-day nationwide strike that was followed by public sector nurses who launched their own 150-day strike. Clinical officers, who are mid-level physician-assistant clinicians in Kenya [14], launched their own 20-day strike led by their union in the midst of the nurses’ strike.

Several studies in Kenya find that strikes are associated with significant decreases in inpatient admissions and outpatient visits in public health facilities [12, 15], but whether strikes lead to increases in population-level mortality is less clear [16–18]. The impact of strikes on maternal and child health services and outcomes has not been adequately explored. One study showed evidence of decreased immunization rates among children at public hospitals during strikes by Kenyan health care workers [19] while another found that rates of self-reported ANC visits and delivery in a health facility were significantly lower in 2017 when there were mass strikes compared to the following year when there were not major strikes [20]. Moreover, strikes may have equally important impacts on care and health systems in ways that are difficult to quantify, including important relational aspects of care between communities, health providers and other health system actors [21].

To investigate the impact of the prolonged strikes by health care workers in Kenya in 2017 on maternal and child health care and services, we conducted a qualitative study using in-depth interviews and focus group discussions (FGDs) with women who were pregnant during strikes in 2017, community health volunteers (CHVs), and health facility managers in one county in western Kenya.

## Methods

### Study setting

This study took place in Trans Nzoia County in western Kenya. Trans Nzoia has a population of approximately one million people with a largely rural and agricultural economy, and generally scores below national averages on primary and maternal child health indicators such as ANC, facility deliveries, and child immunizations [22]. In the most recent Kenya Demographic and Health Survey, the percentage of women who delivered in a health facility was 41.5% in Trans Nzoia compared to 61.2% nationally [4]. According to the Kenya Ministry of Health Master Health Facility List, Trans Nzoia has 182 registered health facilities, which includes seven public hospitals, five private hospitals, one faith-based hospital, and one County referral hospital [23]. In addition to national-level strikes by health care workers, public sector nurses in Trans Nzoia launched their own 44-day strike across the County from February 1, 2017 to March 23, 2017 to protest delays in salaries, remittance of statutory deductions, and promotions [24]. This meant that in Trans Nzoia either physicians, nurses, or clinical officers were on strike in the public health sector for a total of 232 days in 2017.

### Study design and participants

We conducted a qualitative study using in-depth interviews and FGDs with three types of participants. Women who were pregnant in 2017 were invited to participate in FGDs. Eligible women who were pregnant in 2017 were participants in a different study called *Chamas for Change*, a cluster randomized controlled trial to evaluate the effectiveness of a CHV-led, group-based model of care to improve maternal and child health in Trans Nzoia County in western Kenya. At the time of enrollment into the *Chamas for Change* study, participants were 18 years of age or older, pregnant, and less than 24 weeks gestation based on last menstrual period. Additional details on the parent study are provided elsewhere [25]. Using contact information provided for the *Chamas for Change* study, we contacted women by phone or in person with a CHV to see if they would be interested in participating in this study. Purposive sampling was used to ensure women in both peri-urban and rural communities were invited to participate in FGDs.

CHVs and health facility managers were also recruited using convenience sampling for FGDs and in-depth interviews, respectively. Recruited CHVs were volunteers (i.e., they did not receive salaries) who were nominated by their community, worked under the supervision of a facility-based community health extension worker, participated in the *Chamas for Change* training program, and were serving as a CHV in Trans Nzoia County in 2017. Recruited health facility managers were in management-level positions at either public sub-county hospitals, the County referral hospital, or private hospitals in Trans Nzoia in 2017, and were significantly involved in the management and coordination of maternal and child health services at the facility or community level.

### Data collection

Data were collected between March and July 2019. All participants provided written informed consent to participate. In-depth interviews and FGDs were conducted by trained study research assistants in English or Kiswahili and audio recorded. Interviews lasted between 30 minutes and one hour and FGDs lasted between two and three hours, with six to ten participants per FGD. Facilitators used a semi-structured guide. For FGDs with women who were pregnant in 2017, a semi-structured guide asked women to recount their pregnancy journey in 2017 and any challenges or barriers they faced in accessing care. For CHVs and facility managers, semi-structured guides probed experiences delivering maternal and child health care during 2017, any disruptions to care, and strategies to cope with disruptions. Interviews and FGDs inquired about “everyday” and non-strike related barriers to and disruptions in care as a way to better differentiate between barriers and disruptions that were strike-specific versus ones that were unrelated to or exacerbated by strikes. Questions specifically about strikes and their impact on maternal and child health care and services were left to the final section to allow discussions of strikes to emerge inductively. Demographic characteristics of study participants were collected prior to the start of interviews and FGDs.

### Analysis

Audio-recorded interviews and FGDs were transcribed and translated into English (if necessary) by study research assistants. Transcripts were imported into MAXQDA (Version 18.0, VERBI GmbH, Berlin, Germany) for preliminary coding by two investigators (MLS and LYM). Content analysis of data followed a thematic framework approach with preliminary coding using both deductive and inductive approaches. An initial set of codes and themes were defined with additional codes and themes developed through review and re-review of transcripts with team members (JEI, AJ, GA, JCK, and SC). Team members were deeply knowledgeable about maternal and child health care and services in this community having worked in maternal and child health programming for many years. Illustrative quotes of major themes were identified; quotes are included in-text and in a table.

## Results

Seventy-three individuals participated in the study, including 43 women who were pregnant in 2017, 22 CHVs, and eight health facility managers. Participants’ demographic characteristics are provided in Table 1. Both CHVs and facility managers were predominately female (80%). Five health facility managers were nurses, two were public health officers, and one was a community health extension worker (a formal, paid position based at a facility that includes managing a team of CHVs and is usually trained as a nurse or public health officer), with five working in public facilities and three working in private facilities in 2017. CHVs had a median of 11 years of experience (range seven to 30 years) while health facility managers had a median of four years of experience (range two to 33 years) in their position.

**Table 1:** Study participant characteristics

	Women pregnant in 2017 (***n***=43)	CHVs (***n***=22)	Health facility managers (***n***=8)
**Median age, years** (range)	28 (17-43)	46 (34-60)	33 (29-58)
**Sex**, n (%)			
Female	43 (100%)	17 (77%)	7 (88%)
**Marital status**, n (%)			
Single Married	9 (21%) 34 (79%)	- -	- -
**Employment**, n (%)			
Self employed Employed Unemployed	14 (32%) 6 (14%) 23 (54%)	- - -	- - -
**Occupation**, n (%)			
Nurse Public health officer CHEW	- - -	- - -	5 (63%) 2 (25%) 1 (12%)
**Education**, n (%)			
Pre-primary, none Primary Secondary College/Graduate	2 (5%) 17 (40%) 16 (37%) 8 (18%)	0 (0%) 11 (50%) 8 (36%) 3 (14%)	- - -
**Median years of experience** (range)	-	10.5 (7-30)	4 (2.5-33)
**Type of facility employed**, n (%)			
Public Private (faith-based)	- -	- -	5 (63%) 3 (37%)

The remainder of the results section is organized by the major themes that emerged from interviews and FGDs, including: (1) “everyday” challenges in maternal and child health care in this setting in western Kenya, (2) the experiences of women, CHVs, and facility managers in accessing and/or delivering maternal and child health care during strikes, including the impact of strikes on care and strategies to access or maintain care during strikes, (3) inequities that emerged and that were exacerbated by strikes, (4) impact of strikes on key relationships in the health system, (5) perspectives on the legitimacy of strikes by health care workers, and (6) interventions to maintain maternal and child health care during strikes. Illustrative quotes are included in-text and in Table 2, which is organized by theme and sub-theme.

**Table 2:** Illustrative quotes by theme

**Theme 1. “Everyday” challenges in maternal child care**	
Sub-theme 1.1. Cost of care	*“Money can deter one not to go to hospitals. The strike may not be there, but most mothers will still opt to give birth at home because they do not have transport to take hospital… You know money is everything and without it you cannot do anything.”* -FGD with women, Group 1, Participant 8 *“We have challenges within the health sector and at the hospitals whereby as CHVs we refer women, and they get charged Ksh 500 (USD $5) or Ksh 1,000 (USD $10), yet they have Linda Mama insurance to cover both her health and that of the child. So, when I send a mother to deliver [in a public health facility] and she is charged, they say that I am lying and that they are actually asked for money when they go there... If you refer a person and [the health worker] asks for money [from the patient], yet I told them that the service is free. From there, the person will lose respect for you as a CHV.”* *-*FGD with CHV, Group 2, Participant 3
Sub-theme 1.2. Acceptability of care	*“When I was in labor pains, [a health worker] abused me badly and even went ahead and slapped me! Imagine with that condition someone treating you like that? They should be sensitized on how to handle expectant mothers. When I gave birth to my fist born, I concluded I will not be going to hospitals and instead I will give birth in my home.”* -FGD with women, Group 1, Participant 7 *“When we refer [women] to clinics, we require the nurses to treat them in a nice way so that they can be motivated to attend all ANC appointments and even deliver in a facility. When they are harassed and abused verbally, they are discouraged and their attendance drops. It also discourages us as CHVs because by the time you have convinced a woman into accepting that she is pregnant and going to the clinic, you would have done a great job that should not be in vain.”* -FGD with CHV, Group 3, Participant 6
Sub-theme 1.3. Quality of care	*“Because of the shortages, some of the services have not been rendered effectively, and then also the quality of care to the patient is not up to standard…There is no way one nurse can manage a ward and be able to manage all the needs of the patients in that ward…You know when a patient comes [they] expect to get the best but because of these challenges…there are patients who don’t feel like they are satisfied [with] the services.”* -Interview with facility manager, Participant 3
**Theme 2. Experiences of maternal child health care during strikes**	
Sub-theme 2.1. Poor pregnancy outcomes during the strike	*“Pregnant women and children lost their lives. Many women lost their lives during delivery and this strike will be on our memories for a long time to come.”* -FGD with women, Group 2, Participant 8 *“A certain woman delivered at home with the help of a traditional birth attendant and she delivered a healthy baby. Unfortunately, she developed complications during delivery and she died. I was pregnant at the time and I was really worried that I would die during delivery but I thank God I delivered safely. During the strike women go through a lot of challenges.”* -FGD with women, Group 2, Participant 3 *“The strike really affected people from my community, especially the pregnant mothers because maybe she attended her first ANC visit but when she came back for her second, there was the strike. So, she didn’t come for the third, and she defaulted and never met the fourth ANC visit… There were mothers supposed to deliver in the facility accompanied by their birth attendant, but as soon as they heard of the strike, they opted to deliver at home and [this] really affected [their] health.”* -Interview with facility manager, Participant 1
Sub-theme 2.2. Strategies to access care during the strike	*“I also went through such an experience [during the strike] at the [traditional birth attendant] because one needs to be assisted. Sometimes the place can be dirty and she lacks equipment, but you just persevere because you need the help...I was very happy because she did not ask me for any fee and she did not harass me for coming at night. I thanked her very much.”* -FGD with women, Group 3, Participant 1 *“When I arrived in the hospital I only found the watchman who told me that there was a doctor and he went to wake him up. The doctor came, checked up on me, [and] told me that I will give birth at 7:30am. He opened the maternity ward for me and asked me to wait until then. When 7:30am arrived, the doctor came and gave me a condition that in order to treat me, I had to cough up Ksh 2,500 (~USD $250) because doctors were on strike. I told him we had the money as long as he helps me give birth with ease. After he helped me, I paid him the money. It was a government hospital, which we are not supposed to pay any amount since maternity services are for free, but I had to pay because of strike.”* -FGD with women, Group 1, Participant 2 *“I had to take the responsibility of taking her to a Mission hospital [during the strike], but I was told by the doctor in charge that there were no free services and we had to pay at least some money. Since I wanted to help her, I had to use my own money so that she could deliver safely… I had to help her because the community trust in me. When I help them in good and bad times, they will continue trusting in me just the way she did.”* -FGD with CHV, Group 1, Participant 2 *“Yes, we were overwhelmed. We also had to discharge most of our clients earlier than expected because we usually observe a mother for 24 hours after a delivery to ensure both the baby and the mother are stable. But we had to shorten this time to save space for those already in the queue because they kept streaming in.”* -Interview with facility manager, Participant 8
Sub-theme 2.3. Impact on health care workers	*“I really suffered as a person because there was so much suffering, and [patients] come to look at you, you are to solve everything…So me, personally I could not even sleep.”* -Interview with facility manager, Participant 5
**Theme 3. Strike-related inequities in maternal child health services**	
Sub-theme 3.1. Indirect economic impact of strikes	*I spent four thousand shillings when I went to give birth. I sold the maize I had until I didn’t have food at home…Yes, I sold the food I had to pay the hospital bills.”* -FGD with women, Group 2, Participant 4 *“The strike came and even created poverty in the community because now the person is sick, they take their land and they lease, they lease the land even when the person lives there, there is no food at home, children are unable to go to school. This thing largely affected [the community].”* -FGD with CHV, Group 2, Participant 2
Sub-theme 3.2. Vulnerability of poor, especially rural poor, communities	*“It was hell in the community…Because most people affected are the common ones, the poor ones, the common “mwananchi,” [they] are the most affected because you will find the fairly well-off people are able to access services elsewhere. But now you find the local community suffers the most,”* -Interview with facility manager, Participant 8 *“The person that really suffers is the one based in the community because if you look at it, for us that come from the community these private clinics are not many compared to those in towns. For a mother that comes from the village, in order for her to get to the private clinic it will really cost her a lot and life here in the village is difficult. You can’t compare a mother from the village and the one from town because the one in town can easily access the private clinics during strikes as there are plenty of them, so the one being affected is the one from the community.”* -FGD with women, Group 4, Participant 10
Sub-theme 3.3. Role of CHVs in health system	*“Sincerely speaking, the CHVs have been abused. For example, I have been working as a CHV for the past 30 years, I have been working under the health sector, but I have never seen the benefits of this work, yet I have not been employed. I am just doing it out of heart…So, what I am saying is, don’t let me be your stepping stone yet you don’t want to help me.”* -FGD with CHV, Group 1, Participant 6 *“The CHVs are the people who work at the ground and yet those who are paid are in the offices. We walk around villages looking for those who have defaulted medications, and pregnant mothers who need to start their ANC visits... The government should recognize our work as CHVs and they should omit the V in Community Health Volunteer and replace it with the W to be Community Health Workers as it used to be…So, you need to remember us, because we are like your pillars. If we collapse, then you will also collapse.”* -FGD with CHV, Group 1, Participant 2
**Theme 4. Relational dimensions of strikes in the health system**	
Sub-theme 4.1. Impact of strikes on relationships and trust in health system	*“During the strike, I lacked respect and value in the community. When I would visit a household and make a referral, they would not listen to me because there was no one in the facility to provide medical services. They told me I was of no help since the referrals I made were of no use, and I wasn’t providing money for them to attend private facilities. I felt dejected since many people were dying and yet our hands were tied. They were associating us with the doctors and claimed that we were also on strike. This caused an injury to the relationship we had with the people in the community since we are the link between the community and the hospitals. I couldn’t tell the people anything, I had no referrals to make, no drugs to offer; some told me not to step in their houses.”* -FGD with CHV, Group 3, Participant 3 *“When you work in a hospital, you create a relationship with the community…but when there is a strike, they don’t understand why you cannot assist…they tend not to trust us again…It really affected me. We have invested so much in community health…but because of the strike the relationship that we had built was broken, [and mothers] went back to the traditional birth attendants.”* -Interview with facility manager, Participant 7
**Theme 5. Perspectives on the legitimacy of strikes by health care workers**	
Sub-theme 5.1. Support for legitimacy of strikes	*“The doctors were demanding what was rightfully theirs…We saw in the media that the government did not fulfill their end of the bargain.”* -FGD with women, Group 2, Participant 5 *“We could be putting the blame on our doctors, but they are working in unpleasant environments. Most of the times we put our focus on money issues, that the doctors need salary increment, yet they want a good environment at their workplace so that they could perform better and not to be blamed for deaths.”* *-*FGD with CHV, Group 1, Participant 2 *“Sometimes you have to demand for your rights and because now in Kenya, it is a tradition. If you want something you have to go on strike because if you negotiate, you will never get [it]. Going on strike is not a good thing, because let’s say for the nurses and doctors the impact of the strike was too much on the patient, and politicians who are well off go to private facilities. So, it’s like they go on strike, but the politicians can’t feel the pain.”* -Interview with facility manager, Participant 4
Sub-theme 5.2. Lack of support for legitimacy of strikes	*“The advantage of the strike is only felt by the doctors because they don’t lose their loved ones. They can treat them at home or even send them abroad for medication since they have money. The disadvantage of [the] strike is felt by us because we lose a lot of people in the community and there is nothing more painful than seeing someone fighting for their life and succumb before your own eyes…When the strike ends, the doctors return to work but the pain of losing someone because doctors decided not to save lives is a wound that never heals.”* *-*FGD with CHV, Group 3, Participant 4 *“I recommend that the government should investigate those doctors who have their private clinics waiting for strikes and then they recommend patients to go there [during strikes in the public sector].”* -FGD with women, Group 3, Participant 4
**Theme 6. Interventions to maintain maternal child health care during strikes**	
Sub-theme 6.1. Maintenance of minimum services	*“I would ask the government, whenever the nurses working in maternity [wards] want to go on strike or whenever the strike is on, then they should consider salary increments for them so that they can help the expectant mothers and babies. Because when these babies are born, no one knows they can end up being presidents or even doctors. We should not lose lives. So, they should have a salary increment before the strike. And the nurses should take their grievances and complaints before striking so that the government can sort them as they continue working. They should never leave us stranded on the roads.”* -FGD with women, Group 4, Participant 7 *“I think the government could have prioritized pregnant women because strikes don’t normally last long so maternity services should be availed throughout as it might lead to death of the child or mother or both…the maternity wing of the hospital should always be open.”* -FGD with women, Group 4, Participant 12
Sub-theme 6.2. Provide free services in public and private hospitals and clinics	*“I can say these clinics should be free whether [they are] public or private facilities. The services you get in public should also be offered in the private facilities. Any test that is done in public [facilities] for free should also be free in private facilities…pregnant women should be recognized anywhere, both in public and private.”* -FGD with women, Group 3, Participant 5
Sub-theme 6.3. Coordination with private and non-profit sectors during strikes	*“Please work hand in hand from the community, even the private and the faith based [facilities] with public institutions [and] health facilities, so that in case there is another strike in the future…it is good to work hand in hand so that when there is a strike it is good to support these private people to work so that they may fill these gaps.”* -Interview with facility manager, Participant 5 *“I would like to say this Beyond Zero [organization] is all about mothers and children. The government should look for qualified nurses from Beyond Zero so that in cases of strikes they are available and are deployed in every facility, so that when the mothers come at least they can be attended to easily. They can be employed by Beyond Zero and be like the Flying Doctors [organization]. So, when the Beyond Zero vehicle comes it should come with nurses on board to be dispersed in every facility to save these mothers.”* -FGD with women, Group 4, Participant 1
Sub-theme 6.4. Corruption	*“The issue of corruption has really been on a rise. You find that money for paying workers is released by the national government to the County government. But the County government diverts the money. Those giving services in these institutions [i.e., health workers] have children in schools and also some need to pay their rents…yet the money is delayed up to the 15*^*th*^*day of the money. So, corruption has also been a factor that contributes to strikes.”* -FGD with CHV, Group 1, Participant 7

### “Everyday” challenges in maternal and child health care

Participants identified several important barriers to maternal and child health care not related to strikes, including cost of care, long distances to and wait times at facilities, understaffing and drug stockouts, and disrespectful maternity care. Decision making related to facility-based care was complex with women making tradeoffs between care at facilities that they thought was of higher quality, particularly in emergencies, and care from traditional birth attendants that was more likely to be compassionate, confidential, and less expensive. Another challenge to maternal and child health care was confusion about costs of services and what services were covered under new initiatives like Linda Mama. In many cases it was unclear to women and even CHVs whether fees charged for various maternal and child health services were legitimate or whether they were informal charges or even bribes to providers. These “everyday” challenges were important factors in shaping care seeking decisions and quality of care, and they existed apart from issues of strikes.

### Experiences of maternal and child health care during strikes

The majority of participants identified strikes by health care workers as the most significant barrier to maternal and child health care in 2017. Women and CHVs often did not differentiate between health care worker cadres when speaking of strikes, however, there was an understanding that maternal and child health services were “nursing” duties and were generally not performed by other cadres of health care workers even when nurses were on strike. This made pregnant women especially vulnerable during nurses’ strikes, with several CHVs and health facility managers referencing spikes in maternal and child deaths and mother-to-child transmission of HIV due to decreased utilization of ANC. Overall, most participants agreed that pregnant women during the strike were significantly less likely to access adequate ANC, were less likely to deliver in a health facility, and that women who did were more likely to deliver in private facilities. Some women’s experiences during pregnancy were deeply traumatic and chaotic, including several stories of family members or friends dying in childbirth or losing their child during the strike. As one woman said of her community’s experiences during the strikes, *“[they] will be in our memories for a long time to come,”* (FGD with women, Group 2, Participant 8). CHVs and health facility managers noted the psychological toll the strikes took on health care workers as well, with many frontline workers empathizing with the suffering they knew was happening including in their own communities due to the strikes.

There were no coordinated strategies at the County or national level that participants identified to keep maternal and child health services in the public sector operating during the 2017 strikes or to make services in the private sector accessible. Participants described ad hoc efforts often led by individual health care workers, CHVs, and by communities themselves. For health facility managers, this included waiving fees and hiring more staff at private (mostly faith-based) facilities, coordinating services, referrals, and supplies between public and private facilities, and delivering services at public facilities in secret or outside of the facility. For communities and CHVs, this often meant raising money or using their own money to pay for services for family members, friends, and community members.

### Strike-related inequities in maternal and child health services

Strike-related barriers to care were not equitably distributed across communities, and participants highlighted that it was poorer women and children who were most negatively affected by strikes since they were less likely to have the resources to access care in private facilities where most services remained operational. Inequities in terms of the impact of strikes on maternal and child health was a major theme – i.e., that the poor were often left completely without access to care during strikes while a better off minority was largely unaffected because they could pay for care in the private sector. As one facility manager said, it was, *“the poor ones, the common “mwananchi,” [they] are the most affected because you will find the fairly well-off people are able to access services elsewhere.”* (Interview with facility manager, Participant 8). Participants also highlighted the significant economic impact on some patients and communities due to paying for care mostly in the private sector, which had the potential to push families deeper into economic insecurity and poverty. One CHV described strikes as “creating poverty” because sick family members could no longer work, and households spent livelihoods and savings to access care in private facilities. Several CHVs used the opportunity in discussions about strikes by health care workers to highlight their own challenging work environments, that their work was not adequately valued in the health system, and that they should be formally employed and paid by the government. As one CHV said, *“The CHVs are the people who work at the ground and yet those who are paid are in the offices…So, you need to remember us, because we are like your pillars. If we collapse, then you will also collapse,”* (FGD with CHVs, Group 1, Participant 2).

### Relational dimensions of strikes in the health system

Another major theme that emerged was how strikes impacted key relationships in the health system, including patient-provider and community-CHV relationships. Health facility managers and CHVs spoke at length about their efforts to build relationships and trust with communities that were essential to improving maternal and child health services and affecting decisions about care seeking, particularly for pregnant women. Strikes by health care workers strained and sometimes severed these relationships between the community and health system. As one facility manager put it, *“We have invested so much in community health…but because of the strike the relationship that we had built was broken, [and mothers] went back to the traditional birth attendants,”* (Interview with facility manager, Participant 7). CHVs felt they were put in impossibly difficult situations during strikes, receiving little guidance from their supervisors about what services remained open and directions for referring patients to care. Several CHVs described stories of using their own money to help pregnant women and mothers access care during the strike. As one CHV put it in reference to arranging for care at a private hospital for a pregnant mother, “*I had to help her because the community trust in me. When I help them in good and bad times, they will continue trusting in me just the way she did,”* (FGD with CHVs, Group 1, Participant 2).

### Perspectives on the legitimacy of strikes by health care workers

Participants expressed mixed feelings in terms of the legitimacy of strikes by health care workers. Some women and CHVs supported health care workers in their fight for *“what was rightfully theirs”* (FGD with women, Group 2, Participant 3), and believed they were fighting to ultimately improve the public health system and not just serving their own interests. Others were skeptical of striking health care workers for a variety of reasons, including health care workers *“valuing money more than lives”* (FGD with women, Group 3, Participant 3) and suspicions that physicians in particular benefitted because they owned private hospitals and clinics that profited from increased demand during strikes. Among some participants, there was a feeling that health care workers were not as affected by the strike as those in the community, and that *“the advantage of the strike is only felt by the doctors because they don’t lose their loved ones…since they have money,”* (FGD with CHV, Group 3, Participant 4). Several health facility managers acknowledged the pain and suffering caused by strikes while defending the actions of striking health care workers as a last resort, and that *“if you want something you have to go on strike, because if you negotiate, you will never get [it],”* (Interview with facility manager, Participant 4).

### Interventions to maintain maternal and child health care during strikes

Health facility managers and CHVs alluded to several strategies that could mitigate the negative impact of strikes particularly for maternal and child health services. Several health facility managers suggested more formal links between public and private health facilities be established, and that this coordination could help in sharing resources across institutions and referring patients during times of crisis like strikes. CHVs advocated for more training and coordination so that they could better support their communities during strikes alongside demands for salaried positions as formal actors in the health system. Additional strategies to prevent future strikes included policy reforms to revert management of human resources back to the national government and addressing issues of corruption in the health system. Many women felt that the government had a special obligation to certain vulnerable populations, including pregnant women and children, to maintain minimum services during strikes. As one woman said, “*when these babies are born, no one knows…they could end up being presidents or doctors, and we should not lose lives [during strikes]*,” (FGD with women, Group 4, Participant 7). Suggestions for maintaining these services included waiving fees in private facilities and coordinating with non-governmental organizations who could provide health care workers and services during crises like strikes.

## Discussion

Women who were pregnant, CHVs, and health facility managers in Trans Nzoia, Kenya revealed various ways in which the health care workers’ strikes in 2017 impacted maternal and child health care and services in western Kenya. First, strikes in the public sector increased existing barriers to and delays in basic ANC and primary care as well as emergency care during pregnancy that were disproportionately experienced by the poor. While this study took place in only one County, our qualitative findings are supported by studies elsewhere in Kenya that show strikes by health care workers are associated with significant declines in inpatient and outpatient admissions in public health facilities [12], and utilization of services like routine infant immunizations [19]. Several participants referenced people in their community dying due to strikes. Evidence that strikes increase mortality in Kenya is mixed [16–18], however, studies using only inpatient hospital data to capture mortality will miss deaths that happen in the community, and thus undercount overall mortality [26, 27]. In addition, the long-term effects of interrupted ANC, primary care, and early childhood immunizations may be only understood years or decades later. While assessing long-term effects of the 2017 strikes was outside of the scope of this study, we recognize that they are important areas of further study even though they may be difficult to identify and link to specific strikes actions.

Participants described a generally chaotic situation during strikes in 2017 and a lack of coordinated efforts at the national and County level to maintain basic services for maternal and child health. Health systems resilience is a useful concept for understanding internal and external shocks to health systems [28]. Kruk and colleagues define it as “the capacity of health actors, institutions, and populations to prepare for and effectively respond to crises; maintain core functions when a crisis hits; and, informed by lessons learned during the crisis, reorganize if conditions require it,” [29]. Scholars have used the concept of resilience to analyze various shocks to health systems, including infectious disease outbreaks, natural disasters, conflict, migration, and other political and economic crises [30–34], but it may also be well suited to understanding the capacity of health systems to respond to shocks such as mass health care workers’ strikes [35, 36]. We did not find evidence of systems-level strategies to cope and adapt to  strikes but found that individual facilities, health care workers, CHVs, and communities employed their own strategies, consistent with findings published elsewhere [36]. These strategies were mostly “absorptive” and “adaptive” in nature, meaning they were short-term and limited in nature and less likely to represent “transformative” efforts to fundamentally alter or change the health system. CHVs, however, suggested more transformative strategies, such as paying CHVs and providing more supervision and training that could mitigate some negative impacts of strikes especially for primary and community-based care.

A major theme that emerged in our data was the importance of relational dynamics of maternal and child health care, particularly trust, and the impact strikes had on these critical relationships between communities and the health system in this part of Kenya. There has been increasing attention to the role of trust in health systems, particularly among scholars who understand health systems as inherently social and relational systems [37, 38]. Studies show that trust in providers, including community providers such as CHVs, can lead to a variety of improved patient outcomes such as health behaviors, adherence to treatment, patient satisfaction, and quality of life [39–41], but trust may be particularly important and operate in specific ways in the highly gendered context of maternity care [42]. Many women in our study recounted experiences or stories of poor quality and disrespectful maternity care at facilities not related to strikes, consistent with other studies [43]. Research shows that perceptions of poor quality maternal and child health services among mothers, including long wait times, lack of providers and essential equipment and drugs, disrespectful care, and out-of-pocket payments, represents important barriers to seeking care [44]. In our study, CHVs and health facility managers described investing significant time and energy in building trusting relationships with women to improve utilization of services, but that these efforts were often damaged by health care workers’ strikes. It is unclear what the long-term implications of damage to these relationships between women, health care workers, and the health system, and whether the strikes in 2017 or subsequent strikes will have a long-term impact on women’s decision-making about seeking care and trust in the health system [21]. Notably, health care workers raised many issues related to poor care, including lack of investments in public health systems, as central to their motivations to strike [36].

Finally, strikes by health care workers exacerbated systematic and long-standing inequities in the health system. For pregnant women and others in need of care, this meant that it was disproportionately the poor who struggled most to access health care during strikes, while the financial burden of paying for care in the private sector sometimes resulted in pushing families and communities deeper into poverty. Addressing inequities in access to and utilization of health services is a major challenge in the Kenyan health system [45], and this study reveals the ways in which health care workers’ strikes may further entrench these inequities, both in terms of acute impacts on access to services during strikes as well as overall trust in these services that affect care seeking in the future. The 2017 strikes occurred as the government was rolling out a universal maternal and child health policy, Linda Mama, and while health care workers largely support universal health initiatives in Kenya, they feel unsupported and ill-equipped to implement these policies on the ground [46]. Additionally, in this study, CHVs articulated their own precarious working conditions in the health system as unpaid but critically important actors when speaking about health care workers’ strikes. As debates about formalizing community-based health care workers in the health system continue [47], the potential for organizing and collective action among this cadre for better labor protections and decent work may become a new frontier of labor action [48].

There are several limitations to this study. We recruited participants from a single County in western Kenya, and our findings may not be generalizable to other parts of Kenya. Devolution of health services has led to significant autonomy among Counties in managing health services and health workforces, including on issues related to strikes, and it is possible that maternal and child health services were impacted differently across Counties. In addition to national level strikes, health care workers in Trans Nzoia County where this study was based led County-wide strikes, potentially creating additional challenges to maternal and child health services compared to other Counties in 2017. Our study took place about one and a half years after the 2017 strikes by health care workers and there is a risk of recall bias. In addition, the 2017 strikes by health care workers were major social and political events in Kenya with substantial media coverage, which may have created certain narratives. Facilitators of FGDs and interviews, who were familiar with the community and region, were trained to let discussions about strikes emerge inductively from questions about barriers to and disruptions in maternal and child health care. Finally, participants often did not specify which health care workers’ strike they were referring to, so it was difficult to tease out how maternal and child health may be affected differently depending on what cadre of health care worker is on strike.

## Conclusion

In this qualitative study, participants in one County in western Kenya described significant impacts on maternal and child health care and services during the 2017 strikes by health care workers as well as negative economic impacts on households and strained relationships and distrust in the health system. Strikes exacerbated existing and fundamental inequities in the health system, and our findings suggest that addressing these inequities and rebuilding relationships and trust that undergird the health system will be critical to improving maternal and child health care and initiatives during and outside of strikes by health care workers.

## Data Availability

The data generated for the study is not publicly available. Requests for the qualitative analysis codebook and/or de-identified transcripts can be made to the corresponding author.
